# Karonudib has potent anti-tumor effects in preclinical models of B-cell lymphoma

**DOI:** 10.1038/s41598-021-85613-8

**Published:** 2021-03-18

**Authors:** Morten P. Oksvold, Ulrika Warpman Berglund, Helge Gad, Baoyan Bai, Trond Stokke, Idun Dale Rein, Therese Pham, Kumar Sanjiv, Geir Frode Øy, Jens Henrik Norum, Erlend B. Smeland, June H. Myklebust, Thomas Helleday, Thea Kristin Våtsveen

**Affiliations:** 1 Department of Cancer Immunology, Institute for Cancer Research, The Norwegian Radium Hospital, Oslo University Hospital, Ullernschausseen 70, 0379 Montebello, Oslo Norway; 2 KG Jebsen Centre for B Cell Malignancies, Faculty of Medicine, University of Oslo, Oslo, Norway; 3 Department of Oncology and Pathology, Science for Life Laboratory, Karolinska Institutet, Stockholm, Sweden; 4 Weston Park Cancer Centre, Department of Oncology and Metabolism, University of Sheffield, Sheffield, S10 2RX UK; 5 Department of Radiation Biology, Institute for Cancer Research, Oslo University Hospital, Oslo, Norway; 6 Department of Tumor Biology, Institute for Cancer Research, Oslo University Hospital, Oslo, Norway; 7 Department of Cancer Genetics, Institute for Cancer Research, Oslo University Hospital, Oslo, Norway

**Keywords:** Cancer, Drug discovery, Immunology

## Abstract

Chemo-immunotherapy has improved survival in B-cell lymphoma patients, but refractory/relapsed diseases still represent a major challenge, urging for development of new therapeutics. Karonudib (TH1579) was developed to inhibit MTH1, an enzyme preventing oxidized dNTP-incorporation in DNA. MTH1 is highly upregulated in tumor biopsies from patients with diffuse large B-cell lymphoma (DLBCL) and Burkitt lymphoma, hence confirming a rationale for targeting MTH1. Here, we tested the efficacy of karonudib in vitro and in preclinical B-cell lymphoma models. Using a range of B-cell lymphoma cell lines, karonudib strongly reduced viability at concentrations well tolerated by activated normal B cells. In B-cell lymphoma cells, karonudib increased incorporation of 8-oxo-dGTP into DNA, and prominently induced prometaphase arrest and apoptosis due to failure in spindle assembly. MTH1 knockout cell lines were less sensitive to karonudib-induced apoptosis, but were displaying cell cycle arrest phenotype similar to the wild type cells, indicating a dual inhibitory role of the drug. Karonudib was highly potent as single agent in two different lymphoma xenograft models, including an ABC DLBCL patient derived xenograft, leading to prolonged survival and fully controlled tumor growth. Together, our preclinical findings provide a rationale for further clinical testing of karonudib in B-cell lymphoma.

## Introduction

The use of chemo-immunotherapy has greatly improved survival in B-cell lymphoma patients, but relapsed/refractory disease still remains a prominent clinical challenge. Diffuse large B-cell lymphoma (DLBCL) is the most common type of aggressive non-Hodgkin lymphoma and 60–70% of patients are cured with standard treatment with R-CHOP^1^. However, only 10% of patients with relapsed/refractory disease will reach a 3-year progression-free survival^2^. Burkitt lymphoma (BL) is an aggressive lymphoma and requires intensified chemo-immunotherapy^3^, whereas mantle cell lymphoma (MCL) has no standard frontline therapy and is considered incurable with current treatment strategies^4^.

Karonudib (TH1579) is a novel drug developed to target the nucleotide metabolism by inhibiting the nucleotide pool sanitizing enzyme 7,8-dihydro-8-oxoguanine triphosphatase/MutT-homologue-1 (MTH1), encoded by Nudix hydrolase 1 (*NUDT1*)^5^. MTH1 converts oxidized nucleotide triphosphates (8-oxo-dGTP^6^, 2-OH-dATP^7^ and O-6-methyl-dGTP^8^) to the corresponding monophosphate forms. Hence, active MTH1 prevents incorporation of oxidized nucleotides into DNA, which otherwise could cause mispairing, mutations and ultimately cell death^6,9^. Under normal conditions with low reactive oxygen species (ROS) burden, MTH1 is not essential for cell survival^10^. This contrasts cancer cells which frequently upregulate MTH1 to compensate for increased ROS with corresponding higher oxidized nucleotide levels^11,12^. Furthermore, karonudib has a dual mechanism as it also directly and indirectly can perturb microtubule polymerization dynamics via an emerging role of MTH1 in mitosis^13,14^. Inhibition of MTH1 has potent anti-tumor activity in mouse models of colon cancer, malignant melanoma, hepatocellular carcinoma, -lung- and breast cancer^5,15–18^. The first in human clinical trial investigation with karonudib treatment has been initiated for patients with advanced solid malignancies (NCT03036228) and acute leukemias (NCT04077307); https://clinicaltrials.gov.

Here, we tested the effect of karonudib in vitro*,* explored the mechanisms of action and tested the in vivo efficacy of the drug using two different B-cell lymphoma xenograft models. Together, our data demonstrate a potent anti-tumor effect of karonudib and argue for its potential clinical use in treatment of aggressive B-cell lymphoma.

## Materials and methods

### Materials

Karonudib (TH1579) was synthesized in the Helleday laboratory as described earlier^19^.

### Cell lines, human samples and culture conditions

Burkitt lymphoma (BL): BL-41, Raji, Ramos, Rec-1 (Leibniz-Institut-Deutche Sammlung von Mikroorganismen und Zellkulturen (DSMZ)); Germinal center like B-cells (GCB) DLBCL: SU-DHL-6 (DSMZ), SU-DHL-4 (gift from L. Staudt, NCI, USA); Activated B-cell like (ABC) DLBCL: U2932 (DSMZ); DLBCL-double hit: Will-2 (DSMZ); immunoblastic B cell lymphoma: DoHH-2 (DSMZ); mantle cell lymphoma (MCL): Mino, JeKo-1, Granta-519 (DSMZ). Cell lines are kept for up to 8 weeks and mycoplasma tested with Venor GeM Classic (Minerva BioLabs, Berlin, Germany) after 4 weeks and always prior to injection of cells into mice. Cell lines were cultured in RPMI-1640 supplemented with 10% human serum (HS; TCS Biosciences, Buckingham, UK) or 10% fetal calf serum (FCS), penicillin and streptomycin, and maintained at 37 °C in 5% CO_2_. Cell line authentication was done by PCR-single-locus technology for 21 independent PCR systems (Eurofins, Denmark).

Peripheral blood was obtained from anonymous, healthy donors at the Blood Bank (Oslo University Hospital, Norway), with informed consent and approval from regional authorities, Regional Ethical Committee for Medical and Health Research Ethics (REK S-03280). Cells were maintained and activated as described earlier^20,21^. B cells were purified using Dynabeads CD19 Pan B according to manufacturer's instruction (Thermo Fischer Scientific). The B cells were activated minimum 24 h prior to experiments. The BL-41-luc cell line for xenograft studies has previously been described^20^. Patient derived DFBL-49659-V2 cells were obtained from PRoXe (The Public Repository of Xenografts, Dana-Farber Institute of Cancer).

### Cell viability, apoptosis, cell cycle analysis and DNA damage

Measurement of relative cell growth (CellTiterGlo, 72 h, karonudib (0.0625–1 µM)), viability (propidium iodide, 72 h, karonudib (0.25–1 µM)) and apoptosis (Active Caspase-3, 24 h, karonudib (0.5 µM)) was performed as previously described^20^. Proliferation (72 h, karonudib (0.25 µM) was performed using Cell Trace Violet (ThermoFisher Scientific). Terminal deoxynucleotidyl transferase dUTP nick end labeling (TUNEL) was performed together with cell cycle analysis after 6, 12 and 24 h with karonudib treatment (0.5 µM) as previously described^20,22,23^. For cell cycle studies live/dead cell staining (near-IR dead cell stain kit L10119, Thermo Fisher Scientific) was performed prior to fixation. Antibodies: rabbit anti-phospho-histone H3 (pS10 #06-570 1:500; Merck), mouse anti-phospho-γ-histone H2AX (pS139 clone JWB301, #05-635 1:500; Merck), donkey anti-mouse IgG-Alexa488 (#715-545-150 1:500; Jackson Immunoresearch, West Grove, PA), and goat-anti-rabbit IgG-PE (1:500; Thermo). In addition we used biotin-16-dUTP (Merck), streptavidin-Cy5 (PA45001 1:400; GE Healthcare, UK) and Hoechst 33258 (2 µg/ml). Hoechst stained cells were stored at 4°C over night before analysis. Flow cytometry data were analyzed using the online Cytobank flow cytometry software (https://community.cytobank.org)^24^ or FlowJo v10.

### Gene expression profiling

Total RNA was isolated (MiRNeasy, Qiagen, Hilden, Germany) after 12 h treatment with karonudib (0.5 µM). The microarray analyses were performed on GeneChip Human Gene 2.0 ST Array (Affymetrix, Santa Barbara, CA). Two replicates were run per cell line (BL-41 and Mino). Gene set enrichment analysis was performed using the GSEA software v.3.0^25,26^, combining both cell line data against predicted gene sets (Hallmark datasets) downloaded from the MSigDB collection^27^. A thousand permutations were performed to test against control and karonudib treated cells*.* Gene sets with false discovery rate (FDR) *q* values < 0.1 were regarded as significantly enriched gene sets. Microarray data is available at NCBI’s Gene Expression Omnibus with accession number GSE123449. Publicly available mRNA expression data was used from the LLMPP study: Follicular Lymphoma (FL) (*n* = 191) GSE53820^28^, ABC-DLBCL (*n* = 176) GSE10846^29^, GCB-DLBCL (*n* = 97) GSE10846^29^, BL (*n* = 24) GSE4732^30^ and B-cells from PBMC (*n* = 5) GSE46062^31^. Microdissected samples are from GSE12453^32^.

### Western immunoblotting

Cells were lysed and processed for SDS-PAGE, and immunoblotting was performed as described earlier^20,33^. Antibodies: mouse anti-MTH1 (SC-271082; Santa Cruz Biotechnology, TX), rabbit anti-GAPDH (GTX102784; GeneTex, CA), and HRP-conjugated donkey anti-mouse and –rabbit IgG (Jackson Immunoresearch). Full immunoblots are displayed in Supplemental Fig. 7.

### Modified Comet assay

The modified comet assay has been described previously^5,15^. Briefly, BL-41 cells were treated with 0.5 µM Karonudib for 24 h and 20 mM KBrO3 for 2 h. Cells were harvested by centrifugation, mixed with 1.2% low-melting agarose at 37°C and added to fully-frosted slides (Thermo Fisher Scientific). Cells were kept in lysis buffer (2.5 M NaCl, 0.1 M EDTA, 10 mM Tris pH 10.0, 10% DMSO, 1% Triton X-100) at 4°C O/N in the dark. To detect 8-oxo-dG lesions, slides were incubated with recombinant 8-oxoguanine DNA glycosylase (OGG-1) (2.0 μg/ml) diluted in enzyme reaction buffer (40 mM HEPES pH 8.0, 0.1 M KCl, 0.5 mM EDTA and 0.2 mg/ml BSA) at 37°C for 45 min. Electrophoresis was run at 4°C at 300 mA, 25 V for 30 min. DNA was counterstained with SYBR Gold dye (Thermo Fisher Scientific) and images were acquired with a 10× objective in a Zeiss IF microscope and quantified using Comet Assay IV software (Instem, UK).

### Gene editing

Gene editing by CRISPR-Cas9 technology was done by electroporation of validated^34^ guide RNA sequence for MTH1 (CATGAAAAAGCGAGGCTTCG (Integrated DNA technology)) in Mino-Cas9 cells created as previously described^35^.

### Animal experiments

The care and handling of animals for the present study was approved by the Norwegian Food Safety Authority in compliance with the European Convention of the Protection of Vertebrates Used for Scientific Purposes in compliance with the ARRIVE guidelines (Project ID11675). NOD.Cg-Prkdc^scid^ Il2rg^tm1Wjl^/SzJ (NSG) mice were bred in-house. Based on prior experiments with these mice and BL-41-luc cell line, *n* = 11 for each of treatment and control groups were chosen^20^. Mice (6–10 weeks old), were injected subcutaneously with 2 × 10^6^ BL-41-luc cells. Tumor take was measured by bioluminescent imaging (IVIS spectrum in vivo imaging system) after 5 days. The mice were grouped within each cage for either karonudib or control based on tumor size in order to have non-biased, comparable groups. The DFBL-49659-V2 ABC DLBCL patient derived xenograft (PDX) was established from a patient who relapsed after receiving autologous stem cell transplant. The patient had received several prior treatments, including bortezomib and rituximab, R-CHOP and RICE. PDX cells were injected intravenously and treatment started at day 12. Mice were randomized in each cage. Pharmacokinetic studies of karonudib have been performed by Berglund et al.^5^, and the same dosing and treatment regiments that were found to be optimal were used in this study. Briefly, karonudib was dissolved in a 20% (w/v) solution of hydroxypropyl-β-cyclodextrins in acetate buffer (pH 4.6) to a concentration of 9 mg/ml. Mice were given karonudib (90 mg/kg) twice a day (b.i.d) by oral gavage three times a week (Monday–Wednesday–Friday schedule), until all control animals were euthanized due to tumor size in the BL-41-luc model and appearance of clinical signs in the PDX model. Tumor growth was monitored by IVIS^20^ or T_2_-weighted magnetic resonance imaging (MRI) using 7 T MR system (Biospec 70/20 USR, Bruker BioSpin MRI GmbH, Ettlingen, Germany) equipped with a volume T/R resonator with 75/40 mm diameter at regular intervals on days without drug administration. The volume of the spleen was measured by manual delineation of the spleen on the T_2_-weighted images in OsiriX^36^.

### Cellular thermal shift assay

The cellular thermal shift assay (CETSA) has been described previously^5^. CETSA was performed on BL-41-luc cells from the xenografts frozen in LN_2._ Vehicle treated mice were randomized and then given karonudib (90 mg/kg) or vehicle both 18 h and 4 h before euthanization.

### Microscopy

Hoechst stained cells from the flow cytometric cell cycle analysis were also prepared for microscopy using Cytospin as previously described^37^.

### Statistical testing

Statistical significance was determined by two-tailed unpaired Student’s t-test and one-way ANOVA in addition to Log Rank test for the Kaplan–Meier plots in the animal studies. GraphPad Prism was used for calculations. Differences were considered to be significant if *P* < 0.05.

All experiments were approved and performed according to Institutional guideline of Oslo University Hospital. All experimental protocols using animals were approved by the Norwegian Food Safety Authority (Forsøksdyrsforvaltningens tilsyns og søknadssystem, FOTS), which is the licensing committee and the Department of Comparative Medicine, Oslo University Hospital, in compliance with the European Convention of the Protection of Vertebrates Used for Scientific Purposes (Project ID11675). Peripheral blood was obtained from anonymous, healthy donors at the Blood Bank (Oslo University Hospital, Norway), with informed consent and approval from regional authorities, Regional Ethical Committee for Medical and Health Research Ethics (REK S-03280) which is the licensing committee.

## Results

**Figure 1:**
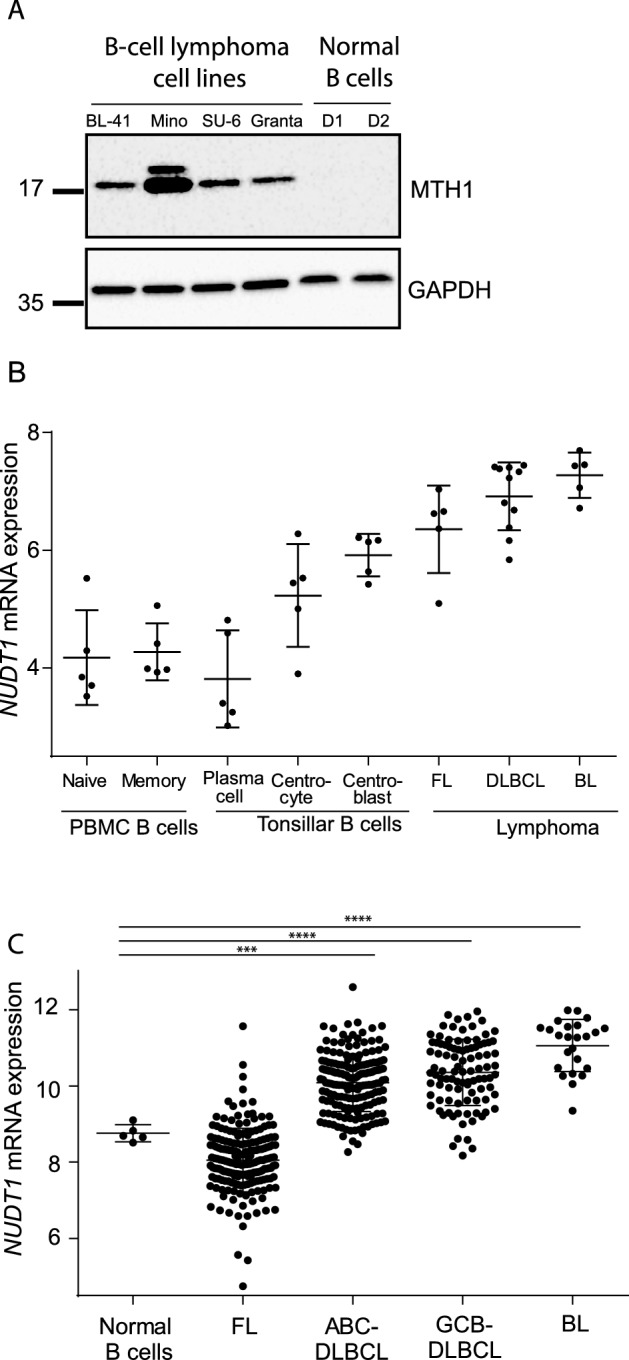
MTH1 is upregulated in B-cell lymphoma cell lines and patient samples. (**A**) MTH1 protein expression in B-lymphoma cell lines and in normal B cells activated with CD40L/IL21 for 24 h (PBMCs, two donors; D1–D2). (**B**) *NUDT1* mRNA data from microdissected samples from subpopulations of B cells from FL (*n* = 5), BL (*n* = 5) and DLBCL (*n* = 11) from Brune et al.^32^. (**C**) *NUDT1* mRNA levels from the LLMPP-database: FL (*n* = 191), ABC-DLBCL (*n* = 176), GCB-DLBCL (*n* = 97), BL (*n* = 24), and B cells from PBMCs (*n* = 5). Stars represent significance compared to normal B cells.

To investigate the relevance of targeting MTH1 protein in B-cell lymphoma, we first investigated protein expression. MTH1 was highly expressed in B-cell lymphoma cells, while it was not detectable in activated peripheral blood B cells from healthy donors (Fig. 1A). Analysis of a published dataset^32^ revealed increased expression of the *NUDT1* mRNA in primary samples from patients with follicular lymphoma, diffuse large B-cell lymphoma and burkitt lymphoma (Fig. 1B). These data also demonstrated highest expression of *NUDT1* in healthy donor germinal centre B cells, in particular centroblasts, whereas the mRNA expression was lower in naive and memory B cells. *NUDT1* mRNA data were also obtained from the Leukemia Lymphoma Molecular Profiling Project (LLMPP) datasets^28–31^, demonstrating significantly higher transcription level of *NUDT1* in ABC and GCB DLBCL subtypes and in BL, but not in FL as compared to healthy donor B cells (Fig. 1C). These results indicate that MTH1 might be a rational target in aggressive B-cell lymphoma.

**Figure 2:**
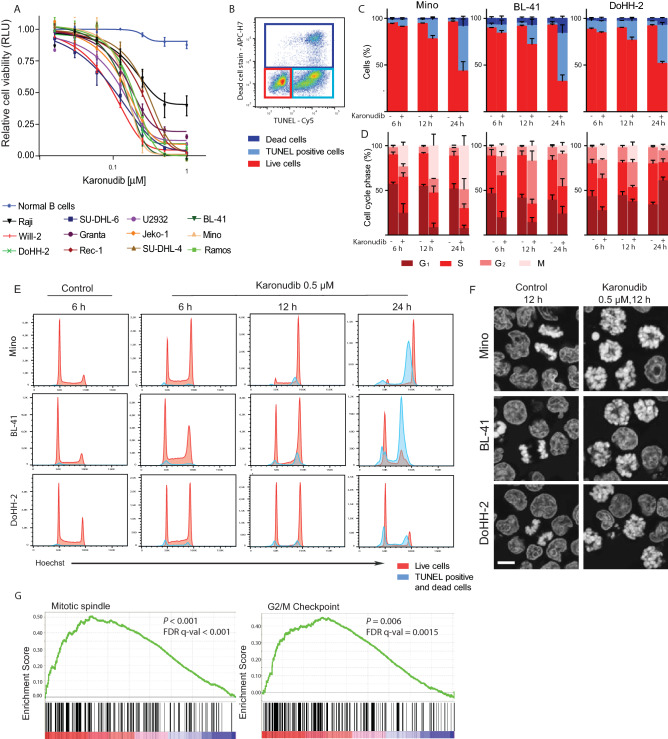
Karonudib induces apoptosis after metaphase arrest in lymphoma cell lines, without affecting healthy B cells. (**A**) Cell viability was measured by CellTiterGlo and normalized to untreated samples (karonudib, 0.0125–1 µM, 72 h, *n* = 3). (**B**) Gating strategy to identify apoptotic cells is shown for Mino cells (karonudib, 0.5 µM, 24 h): TUNEL^+^, light blue gate;, dead cells, dark blue gate; and live cells in red gate, and (**C**) the frequencies of these populations in Mino, BL-41 and DoHH-2 (karonudib, 0.5 µM, *n* = 3). (**D**) Cell cycle analyses of the live cells (from **C**). (**E**) Histograms representing live (red) and dead/apoptotic cells (blue) show G_2_ accumulation and M-arrest after karonudib treatment. DoHH-2 also show a G_1_ arrest at 24 h and cells entering apoptosis from G_1_-arrest. (**F**) Microscopy of Hoechst stained cells from the same experiment in (**B**–**E**) (12 h with or without 0.5 µM karonudib, 63×/1.4 objective, bar = 10 µm). An error in the spindle assembly is clearly visible in cells treated with karonudib (images captured randomly), whereas images of successful chromosome segregation in control cells were chosen to illustrate the difference. (**G**) GSEA of BL-41 and Mino cells (karonudib, 0.5 µM, 12 h), identified Mitotic spindle and G_2_/M-Checkpoint as significant altered gene sets (*P* < 0.001 and *P* = 0.006) (GSEA software v.3.0).

To test if karonudib had potent anti-lymphoma activity in vitro, a dose–response assay was performed in B-cell lymphoma cell lines representing ABC or GCB DLBCL, MCL and BL. Karonudib induced potent inhibitory effect on cell growth in all lymphoma cell lines, with IC_50_ ranging from 0.1 to 0.3 μM (Fig. 2A). In contrast, karonudib showed no effect on normal B cells from peripheral blood, activated with CD40L and IL-21 (Fig. 2A). In lymphoma cell lines, karonudib induced cell death determined by Propidium Iodide staining in a dose dependent manner (Supplemental Fig. 1A). Detection of active caspase-3 after 24 h of karonudib treatment confirmed induction of apoptosis selectively in B-cell lymphoma cells, but not in normal B cells (Supplemental Fig. 1B).

Combination of viability staining with TUNEL assay demonstrated an increase in the fraction of TUNEL-positive apoptotic cells after 12 h of karonudib treatment in three sensitive lymphoma cell lines, with a further increase to > 50% apoptotic cells after 24 h (Fig. 2B, C). Few necrotic cells were detected (dead cell stain ^+^ TUNEL^−^). Hence, apoptosis is the predominant form of cell death after karonudib treatment. The effect of karonudib on cell cycle distribution, cell cycle-resolved DNA damage and apoptosis was then studied in a time dependent manner. Karonudib induced accumulation of cells in G_2_ and particularly M phase for all three cell lines at 6 and 12 h after initiated treatment, while only the Mino cell line had a further increase in the percentage of G_2_ and mitotic cells at 24 h (Fig. 2D). After 24 h treatment DoHH-2 was clearly-arrested in G_1_ (Fig. 2D, E, red histograms). The apoptotic/dead cells (blue histograms) revealed that karonudib induced apoptotic cell death from the G_2_/M phase in cell lines with mutant TP53 (Mino and BL-41, Supplemental Table 1), whereas DoHH-2 (wt TP53) underwent apoptotic cell death also from G_1_ phase (Fig. 2E, blue histograms). It was not possible to assess whether the apoptotic cells originated from G_2_ or mitosis, as phospho-histone H3 expression is lost when mitotic cells become apoptotic (unpublished). The accumulated live cells were mainly mitotic cells as determined by phospho-histone H3 (ɣH2AX) expression (Supplemental Fig. 2B). Microscopic evaluation showed that the cells were arrested in prometaphase due to failure in spindle assembly, with formation of monopolar spindles and lack of chromosome alignment on a metaphase plate (Fig. 2F). Cells in anaphase were not detected among the karonudib treated cells, in contrast to cells grown in the absence of karonudib (Fig. 2F). Together, this indicates that karonudib effectively blocked progression through mitosis. The arrest in prometaphase was not accompanied by increased levels of ɣH2AX (Supplemental Fig. 2C). DNA damage in S phase cells leads to increased replication stress and decreased DNA replication rate^22^. However, karonudib did not increase ɣH2AX in S-phase cells (Supplemental Fig. 2C). Hence, karonudib does not increase the replication stress from the baseline levels, in contrast to what was observed with the PARP inhibitor olaparib^22^. To find the transcriptomic changes induced by karonudib, gene expression profiling was performed of Mino and BL-41 cells treated with or without karonudib for 12 h. Gene set enrichment analysis was performed by combining the two cell lines for analysis, and revealed that only two gene sets were significantly dysregulated after karonudib treatment. These were gene sets annotated as “mitotic spindle” and “G_2_/M arrest” (Fig. 2G; Supplemental Table 2 and Supplemental Fig. 3), which is in accordance with the results from the functional analysis by flow cytometry (Fig. 2D, E).

**Figure 3:**
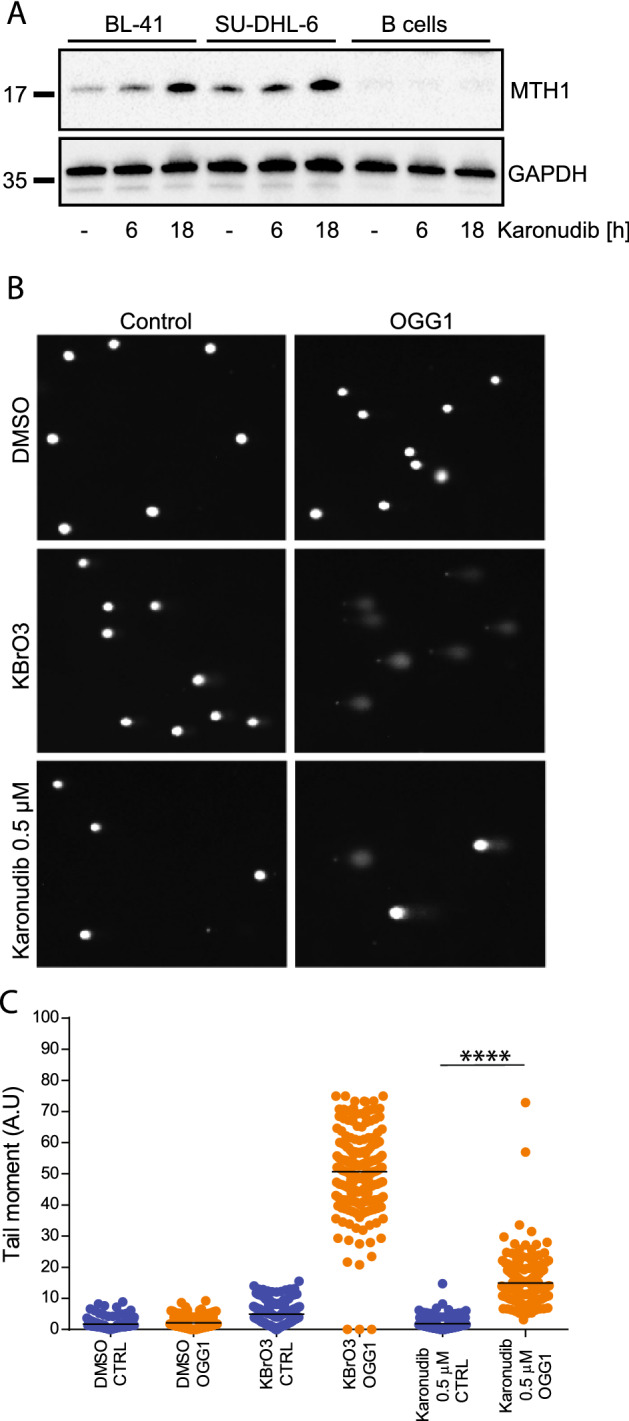
Inhibition of MTH1 leads to increased incorporation of 8-oxo-dGTP. (**A**) MTH1 expression was detected in total cell lysates (karonudib, 0.5 µM, 6 h, 18 h). (**B**, **C**) A modified comet assay was used to indirectly measure incorporation of 8-oxo-dGTP. An increase in comet tails indicates nick in the DNA by recombinant 8-oxoguanin-DNA-glycosylase (OGG-1) where 8-oxo-dGTP is present. BL-41 cells (karonudib, 0.5 µM, 24 h. KBrO_3_ was used as a positive control (*n* = 200)). Images are acquired by Zeiss IF microscope and quantified using Comet Assay IV software.

Karonudib inhibits the activity of MTH1, and also stabilizes the MTH1 protein^5^. To test if karonudib had a similar effect in normal and malignant B cells, the BL-41 and SU-DHL-6 cell lines and normal B cells were treated with karonudib for 6 and 18 h before preparing cell lysates and analysis of protein expression by Western immunoblotting. This revealed that karonudib stabilized MTH1 in the B-cell lymphoma cells, but had no detectable effect in normal B cells (Fig. 3A). Drugs like karonudib that interfere with the MTH1 activity would be expected to increase the incorporation of 8-oxo-dGTP into DNA. This was indeed confirmed by the modified comet assay where BL-41 cells were treated with karonudib and the incorporation of 8-oxo-dGTP was analyzed indirectly by adding recombinant OGG-1 glycosylase (Fig. 3B, C, *P* < 0.001).

**Figure 4:**
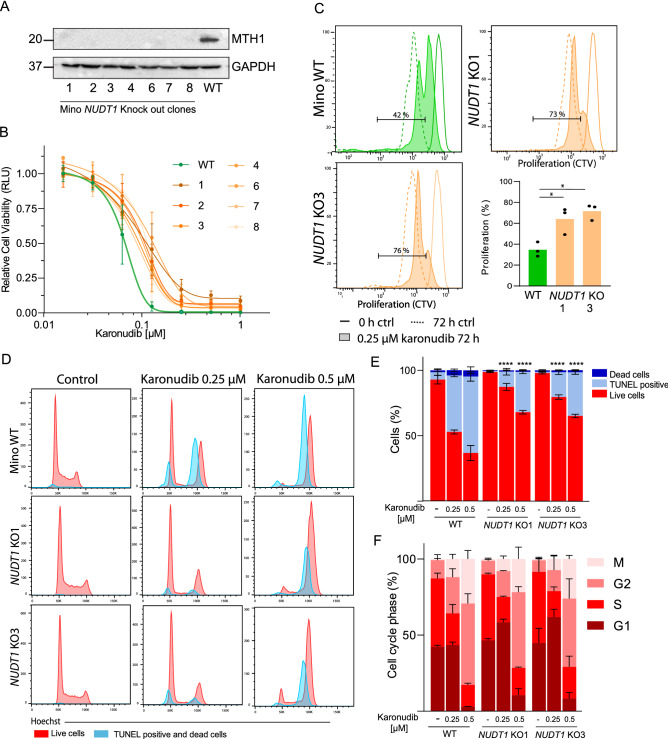
*NUDT1* KO in Mino cell lines confirm dual mechanism of karonudib. Two *NUDT1* KO clones of Mino were used to assess the effect of MTH1 loss in the lymphoma model. (**A**) Western immunoblot of seven clones show loss of MTH1 expression. (**B**) Cell viability was measured in Mino WT (green) and *NUDT1* KO (orange) by CellTiterGlo and normalized to untreated samples (karonudib, 0.0125–1 µM, 72 h, *n* = 3) (**C**) Proliferation using Cell Trace Violet (CTV) is shown for Mino WT, *NUDT1* KO1 and *NUDT1* KO3 (continuous line: control cells at time zero, dashed line: control 72 h and filled histogram: 0.25 µM karonudib 72 h). Quantification of the % (shown in gate) of proliferating cells in WT and 0.25 µM karonudib treated cells. *Significant differences (*P* < 0.05), repeated measurements one-way ANOVA with Dunetts correction between WT and the KO-clones. (**D**) Gating strategy to identify live, dead and apoptotic cells is shown for Mino cells in Fig. 2B. Histogram representing live (red) and dead/apoptotic cells (blue) show G_2_ accumulation and M-arrest after karonudib treatment, 24 h. (**E**) TUNEL^+^, light blue,; dead cells, dark blue,; and live cells in red show the frequencies of these populations in Mino WT, *NUDT1* KO 1 and *NUDT1* KO 3 (karonudib, 0.25 and 0.5 µM, 24 h, *n* = 3). *Significant differences (*P* < 0.0001, one-way ANOVA with Dunetts correction) between WT and the KO-clones for both concentrations of karonudib. (**F**) Cell cycle analyses of the live cells from (**E**).

Because the specificity of the TH588 inhibitor has been debated^34,38^, we generated *NUDT1* knockout (KO) lymphoma cells using a Mino-Cas9 cell line. We successfully generated seven clones that lacked expression of MTH1 (Fig. 4A, Supplemental Fig. 4). Viability and proliferation assays showed an increased tolerance for karonudib in the *NUDT1*-KO clones (Fig. 4B, C). We selected two KO clones (KO1 and KO3) for further testing to gain insight into the mechanisms of karonudib. Both *NUDT1* KOs had significantly higher proliferation in the presence of karonudib as compared to wild type (WT) cells, demonstrating on-target effect of the drug (Fig. 4C). However, the KO cells were not completely resistant to karonudib, hence demonstrating additional MTH1-independent toxic effect of the drug. Cell cycle analysis demonstrated prominent accumulation in G_2_ and mitotic arrest in the two *NUDT1-*KO clones, similar to the WT cells after karonudib treatment (Fig. 4D, F). However, the two KO clones demonstrated increased resistance to karonudib induced apoptosis as compared to WT cells, with 68 and 65% live cells for *NUDT1*-KO1 and KO3 as compared to 37% live cells for WT cells when treated with 0.5 µM of karonudib (Fig. 4E, *P* < 0.0001). Cell cycle distribution was analyzed in the same experiments, and revealed that karonudib had similar effect on cell cycle arrest in the *NUDT1*-KO clones as in the WT cells (Fig. 4F). All together, these experiments demonstrate an on-target effect of karonudib for induction of apoptosis, whereas the cell cycle arrest is independent of MTH1 expression.

**Figure 5:**
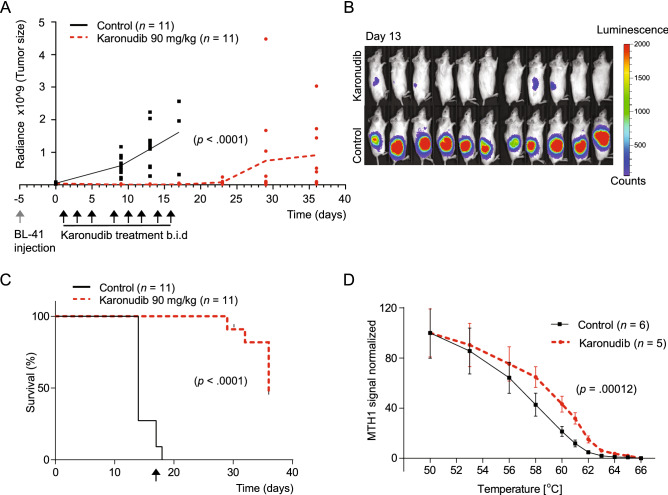
Karonudib has a strong anti-tumor effect in vivo*.* (**A**) Tumor growth of BL-41-luc cells was monitored with IVIS luminescence measurements (physical units of surface radiance (photons/s/cm^2^/st)) at day 0, 9, 13, 17, 23, 29 and 36 after treatment start. Treatment was initiated 6 days after inoculation, and karonudib was given *per os, b.i.d,* 90 mg/kg three times a week for 16 days. Tumor growth was significantly decreased in mice treated with karonudib already at day 9, 13 and 17 compared with treatment start (*P* < 0.0001 for all time points). There was a significant difference (*P* < 0*.*0001) between treatment and vehicle both 9 and 13 days after treatment start (unpaired two-tailed t-test with Welsh's correction). (**B**) IVIS image of animals at day 13. (**C**) Kaplan–Meier is based on tumor size (> 2 cm^3^ or 2 cm length in one direction). Median survival was 14 vs. 36 days in control vs. karonudib treated mice (*P* < 0.0001, Log-rank test). Arrow indicates end of treatment. (**D**) In vivo target engagement of karonudib to MTH1 shown by CETSA. Vehicle treated mice were randomized and then given karonudib (90 mg/kg) or vehicle 18 h and 4 h prior to euthanization and dissection of tumor tissue. Melting curve for MTH1 with a significant T_m_ shift of 2.55°C is shown (*P* = 0.00012, karonudib treated (*n* = 5), vehicle (*n* = 6)) measured at 50% MTH1 denaturation.

To test the efficacy of karonudib in vivo, the aggressive Burkitt lymphoma cell line BL-41-luc was inoculated subcutaneously in NSG mice, and in vivo luminescence was used to confirmed equal tumor load prior to treatment (Supplemental Fig. 5A, B). For mice in the treatment group, karonudib was administered by oral gavage twice daily, 3 days a week over a period of 16 days. A significant decrease in tumor size in karonudib treated mice was observed from day 9 after treatment start (Fig. 5A, *P* < 0.0001). Imaging at day 13 showed a significant difference in tumor load between control and karonudib treated mice (Fig. 5B, *P* < 0.0001). However, about a week after end of treatment, tumor re-growth was observed. All mice eventually showed tumor re-growth, although at the end of study, three of the mice had tumor detectable by luminescence imaging only (Fig. 5A; Supplemental Fig. 5A). The survival curve showed a median time to end point of 14 versus 36 days for control and karonudib treated mice, respectively (Fig. 5C, *P* < 0.0001). To confirm target drug binding in vivo, a Cellular Thermal Shift Assay (CETSA) was performed in tumors from mice treated with karonudib 24 h and 4 h before sampling (Fig. 5D). The melting curve obtained from the CETSA analysis demonstrated a thermal shift of 2.55 °C (*P* = 0.00012), indicating specific binding of karonudib to MTH1 in vivo. This indicated an on-target effect of karonudib upon in vivo treatment.

**Figure 6:**
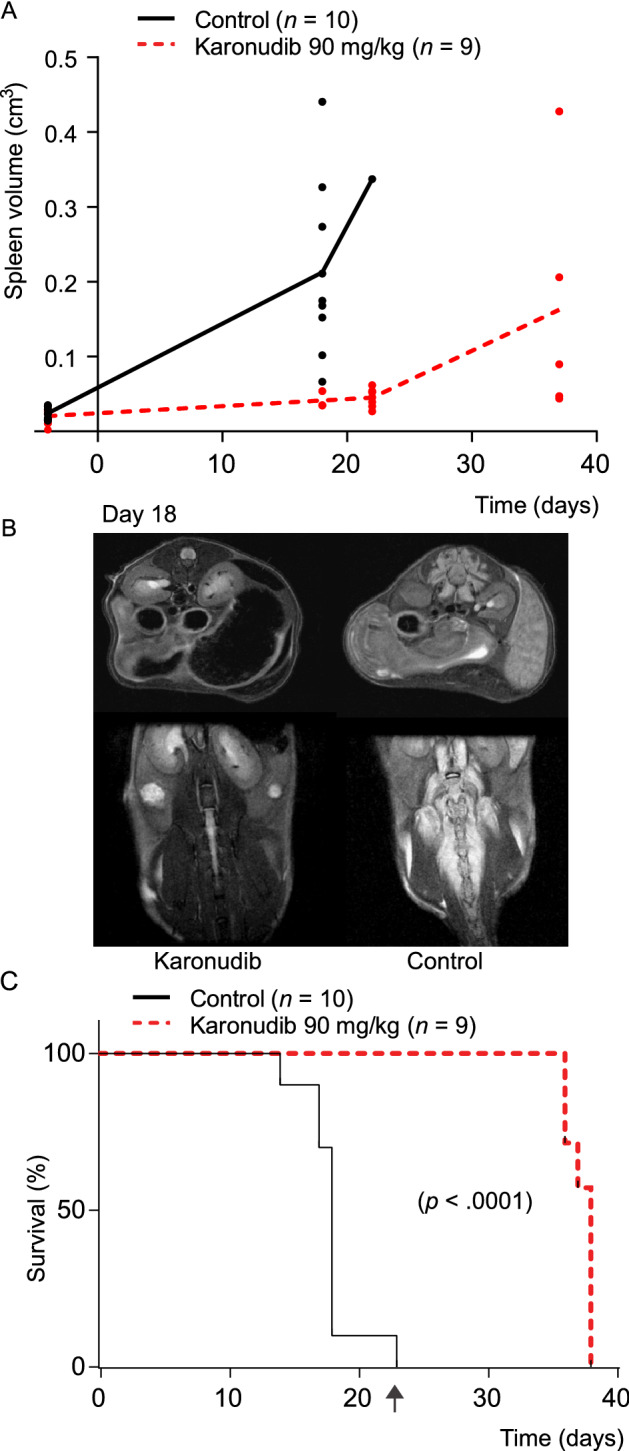
Karonudib has a strong anti-tumor effect in the ABC DLBCL PDX model DFBL-49659-V2. Tumor cells were injected intravenously and tumor growth monitoring of spleen and bone marrow with MR-imaging. (**A**) The treatment was initiated 12 days after tumor injection, and continued for 23 days as indicated (karonudib *per os*, *b.i.d*, 90 mg/kg three times a week). Spleen volume was measured by manual delineation on the T_2_-weighted images. First scan was performed 4 days prior to treatment start up. (**B**) T_2_-weighted MRI scans of a karonudib and vehicle treated mice at day 18. The arrows indicate increased spleen and edema around the spine in the untreated animal. (**C**) Median survival was 18 vs. 38 days in control vs. karonudib treated mice (*P* < 0.0001, Log-rank test). The mice were monitored until they showed clinical signs. Arrow indicates end of treatment. All images are presented in Supplemental Fig. 6A.

To further validate the preclinical effects of karonudib in B-cell lymphoma, we used a patient-derived lymphoma xenograft (PDX) model by intravenous injection of lymphoma cells from an ABC-DLBCL patient into NSG mice. This model demonstrated homing of tumor cells to the spleen and bone marrow, but with very few circulating tumor cells in the blood. Karonudib treatment was started 12 days after injection of tumor cells, and followed the same scheme as described for the BL-41 xenograft model, for a period of 23 days. The tumor growth was monitored by MR-imaging and volume measurement of the spleen. Also in the PDX model, karonudib demonstrated potent anti-lymphoma activity which was clearly shown by the lack of increased spleen size in the karonudib treated mice as long as they were under treatment (Fig. 6A; Supplemental Fig. 6A, B). MRI at day 18 after treatment start demonstrated massive infiltration of tumor cells in the spleen and bone marrow of the control mice, while karonudib treated mice had no sign of tumor cells (Fig. 6B; Supplemental Fig. 6A). The survival curve showed a median survival of 18 versus 28 days for control and karonudib treated mice, respectively (Fig. 6C, *P* < 0.0001). The MTH1 level in the karonudib treated group after tumor re-growth was unchanged compared to the control group (Supplemental Fig. 6C) suggesting that tumor cells after relapse still could be sensitive to karonudib treatment. Karonudib was generally well tolerated as demonstrated by similar weight distribution (Supplemental Fig. 6D). Taken together, karonudib was well tolerated and demonstrated potent anti-tumor activity in aggressive B-cell lymphoma preclinical models.

## Discussion

MTH1 inhibitors have demonstrated tumor suppressive properties in colon cancer, malignant melanoma, liver cancer, small cell lung cancer and breast cancer in mice models^5,15–18^. However, the efficacy of MTH1 inhibitors in hematological malignancies has not yet been reported. In this first preclinical study of B-cell lymphoma, we demonstrate a strong inhibitory effect of karonudib on cell growth in a wide range of B-cell lymphoma cell lines at concentrations not affecting healthy donor normal B-cells. Karonudib mediated anti-lymphoma activity through induction of mitotic arrest, leading to apoptosis. Testing of *NUDT1* KO clones revealed that the apoptosis-induced effect of karonudib partially was mediated by MTH1, whereas the cell cycle arrest was an MTH1-independent effect. We further demonstrated potent anti-tumor efficacy of karonudib in vivo in two different lymphoma xenograft models, including an ABC DLBCL PDX xenograft model.

MTH1 was highly expressed in B-cell lymphoma cell lines compared to normal activated B cells, and was therefore identified as a potential target. Based on this information and the fact that MTH1 has been characterized as a non-essential protein in normal cells within a realistic treatment window^39^, we hypothesized that patients with B-cell lymphoma could possibly benefit from inhibition of MTH1. Analysis of publicly available gene expression data have shown that the *NUDT1* expression increases with proliferation status of the B-cell subtype^11^, and is in concordance with the known high tumor cell proliferation rate in these subtypes. In mice, depletion of MTH1 seemed not to affect their health as MTH1 knockout mice appear healthy and have survival rates comparable to normal mice^10^. The human toxicity profile will be addressed in the on-going first in human, first in class phase I clinical trials with karonudib monotherapy in advanced cancer patients (NCT03036228 and NCT04077307 at https://clinicaltrials.gov).

Karonudib has previously been shown to specifically bind to MTH1^5^, and exposure leads to elevated incorporation of oxidized nucleotides into DNA. This effect could be reversed by overexpressing MTH1, MutT (the bacterial version of MTH1) or by pretreatment with ROS scavenger^7^. Here, we demonstrated direct binding of karonudib to MTH1 in mice. Although all mice treated with karonudib eventually relapsed, the level of MTH1 remained similar to the before-treatment level. This suggests that tumor cells after relapse still could be sensitive to karonudib.

Here, we demonstrated that all B-cell lymphoma cell lines displayed a pronounced mitotic arrest in response to karonudib treatment, due to aberrant mitotic spindle formation. This may be related to the mechanism of action of karonudib, targeting microtubuli^13^. The transcriptional changes induced by karonudib in lymphoma cell lines supports this hypothesis as the only significant enrichment profiles identified were "spindle formation" and "G_2_/M-arrest". In addition to mitotic arrest in all three tested lymphoma cell lines, the *TP53* mutation status influenced the type of cell cycle arrest induced. Whereas karonudib induced G_2_/M arrest in cell lines harboring mutant TP53, DoHH-2 with WT TP53 additionally had a pronounced G_1_ arrest after karonudib treatment. Our finding that karonudib had potent inhibitory effect in lymphoma cells harboring mutated TP53 could be an important discovery, as many drugs used in treatment of relapsed lymphoma patients require a functional TP53 to be effective^40–42^ and TP53 loss or mutation is associated with inferior survival^42–45^. Furthermore, karonudib was also potent in lymphoma cells regardless of the mutational or translocation status of *MYC* (Supplemental Table 1). This is essential since double-hit lymphomas with aberrant expression of MYC and BCL2 has dismal outcome^46,47^. Further studies to evaluate therapeutic effect of karonudib in B-cell lymphoma subtypes including high-risk patients are therefore warranted.

**Figure 7:**
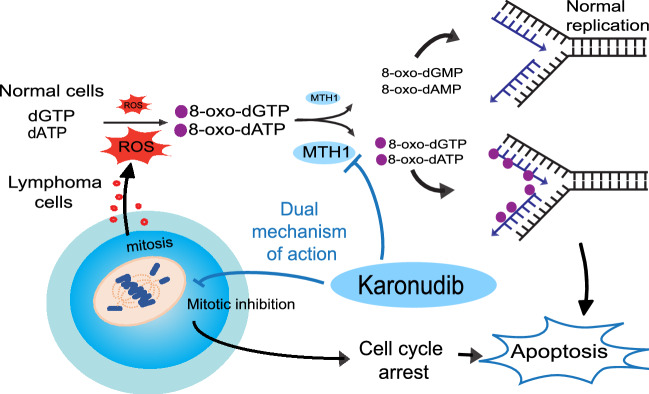
Dual mechanism of karonudib leads to apoptosis in lymphoma cells. Karonudib was developed to target the nucleotide metabolism by inhibiting the nucleotide pool sanitizing enzyme, MTH1. MTH1 converts oxidized nucleotide triphosphates created by reactive oxygen species (ROS) to the corresponding monophosphate forms, preventing incorporation of oxidized nucleotides into DNA. High ROS levels in lymphoma corresponds to high MTH1 levels. Karonudib inhibits the function of MTH1 and results in increased oxidized nucleotides in the DNA, and the drug also perturbs microtubule polymerization and leads to cell cycle arrest and apoptosis.

However, the validity of MTH1 and MTH1 inhibitors in cancer has been debated and TH588, the first generation MTH1 inhibitor, has been shown to bind microtubuli and induce mitotic arrest in MTH1 knockout cell lines^34,38^. Additional mechanism of action has been proposed for the cytotoxic MTH1 inhibitors and indeed karonudib has a dual mechanism of action. Karonudib causes mitotic arrest via disturbed microtubule polymerization and increased oxidized nucleotides incorporated into DNA during mitosis^13^. Interestingly, the non-cytotoxic MTH1 inhibitors can become cytotoxic and elevate incorporation of oxidized nucleotides into DNA when combined with a mitotic arrest compound such as paclitaxel, CENP-E inhibitor^13^, supporting the importance of having a dual mechanism for effective anti-tumor effect. Based on this, we hypothesized that karonudib and TH588 have dual mechanism of action in tumor cells by inhibition of spindle formation during mitosis leading to mitotic arrest, and by inducing increased levels of oxidized nucleotides and increased incorporation of 8-oxo-dGTP in the genome^14^. That drugs have different mechanism of action is however common, even for drugs that have entered clinical trials^48^. Since this has implications for efficacy and toxicity in patients, it calls for stringent genetic validation of action for cancer drugs in the preclinical setting. We generated *NUDT1* KO clones and testing the effect of karonudib. In these clones we demonstrated on-target effect as the apoptosis-inducing effect to the drug was partially lost upon loss of MTH1. However, since *NUDT1* KO clones exhibited a similar cell cycle arrest phenotype as the WT cells after karonudib treatment, this clearly shows that karonudib can induce cell cycle arrest independent of MTH1, and hence has a dual mechanism of action (Fig. 7).

Taken together, our data suggest that karonudib is a new promising drug with a potential broad therapeutic use in B-cell lymphoma. The drug has a dual mechanism of action and may be particular effective in aggressive lymphoma with both high MTH1 levels and high proliferation rate.

## Supplementary Information

Supplementary Information.

## Data Availability

Microarray data is available at NCBI’s Gene Expression Omnibus with accession number GSE123449.

## References

[1] Coiffier B, Sarkozy C (2016). Diffuse large B-cell lymphoma: R-CHOP failure-what to do?. Hematol. Am. Soc. Hematol. Educ. Program.

[2] Gisselbrecht C (2010). Salvage regimens with autologous transplantation for relapsed large B-cell lymphoma in the rituximab era. J. Clin. Oncol..

[3] Casulo C, Friedberg JW (2018). Burkitt lymphoma—A rare but challenging lymphoma. Best Pract. Res. Clin. Haematol..

[4] Maddocks K (2018). Update on mantle cell lymphoma. Blood.

[5] Berglund UW (2016). Validation and development of MTH1 inhibitors for treatment of cancer. Ann. Oncol..

[6] Sakumi K (1993). Cloning and expression of cDNA for a human enzyme that hydrolyzes 8-oxo-dGTP, a mutagenic substrate for DNA synthesis. J. Biol. Chem..

[7] Fujikawa K (1999). The oxidized forms of dATP are substrates for the human MutT homologue, the hMTH1 protein. J. Biol. Chem..

[8] Jemth AS (2018). MutT homologue 1 (MTH1) catalyzes the hydrolysis of mutagenic O6-methyl-dGTP. Nucleic Acids Res..

[9] Yoshimura D (2003). An oxidized purine nucleoside triphosphatase, MTH1, suppresses cell death caused by oxidative stress. J. Biol. Chem..

[10] Tsuzuki T, Egashira A, Kura S (2001). Analysis of MTH1 gene function in mice with targeted mutagenesis. Mutat. Res..

[11] Oda H, Nakabeppu Y, Furuichi M, Sekiguchi M (1997). Regulation of expression of the human MTH1 gene encoding 8-oxo-dGTPase. Alternative splicing of transcription products. J. Biol. Chem..

[12] Fujikawa K, Kamiya H, Yakushiji H, Nakabeppu Y, Kasai H (2001). Human MTH1 protein hydrolyzes the oxidized ribonucleotide, 2-hydroxy-ATP. Nucleic Acids Res..

[13] Gad H (2019). MTH1 promotes mitotic progression to avoid oxidative DNA damage in cancer cells. bioRxiv..

[14] Rudd SG (2020). MTH1 inhibitor TH588 disturbs mitotic progression and induces mitosis-dependent accumulation of genomic 8-oxodG. Cancer Res..

[15] Gad H (2014). MTH1 inhibition eradicates cancer by preventing sanitation of the dNTP pool. Nature.

[16] Einarsdottir BO (2018). A patient-derived xenograft pre-clinical trial reveals treatment responses and a resistance mechanism to karonudib in metastatic melanoma. Cell Death Dis..

[17] Lallo A (2019). Ex vivo culture of cells derived from circulating tumour cell xenograft to support small cell lung cancer research and experimental therapeutics. Br. J. Pharmacol..

[18] Hua X (2019). Karonudib is a promising anticancer therapy in hepatocellular carcinoma. Ther. Adv. Med. Oncol..

[19] Scobie, M. H., Koolmeister, T., Deques, T., Decrose, S., Jacques-Cordonnier, M., Marie-Caroline, J. Pyrimidine-2,4-diamine derivatives for treatment of cancer. Sweden patent WO2014084778 (2014).

[20] Vatsveen TK (2018). Artesunate shows potent anti-tumor activity in B-cell lymphoma. J. Hematol. Oncol..

[21] Rasmussen AM, Smeland EB, Erikstein BK, Caignault L, Funderud S (1992). A new method for detachment of Dynabeads from positively selected B lymphocytes. J. Immunol. Methods.

[22] Rein ID (2015). New distinct compartments in the G2 phase of the cell cycle defined by the levels of gammaH2AX. Cell Cycle.

[23] Landsverk KS, Lyng H, Stokke T (2004). The response of malignant B lymphocytes to ionizing radiation: Cell cycle arrest, apoptosis and protection against the cytotoxic effects of the mitotic inhibitor nocodazole. Radiat. Res..

[24] Kotecha, N., Krutzik, P. O. & Irish, J. M. Web-based analysis and publication of flow cytometry experiments. in *Current protocols in cytometry/editorial board, J. Paul Robinson, managing editor ... [et al.]* Chapter 10, Unit 10 17. 10.1002/0471142956.cy1017s53 (2010). 10.1002/0471142956.cy1017s53 PMC4208272 20578106

[25] Mootha VK (2003). PGC-1alpha-responsive genes involved in oxidative phosphorylation are coordinately downregulated in human diabetes. Nat. Genet..

[26] Subramanian A (2005). Gene set enrichment analysis: A knowledge-based approach for interpreting genome-wide expression profiles. Proc. Natl. Acad. Sci. USA.

[27] Liberzon A (2015). The Molecular Signatures Database (MSigDB) hallmark gene set collection. Cell Syst..

[28] Brodtkorb M (2014). Whole-genome integrative analysis reveals expression signatures predicting transformation in follicular lymphoma. Blood.

[29] Alizadeh AA (2000). Distinct types of diffuse large B-cell lymphoma identified by gene expression profiling. Nature.

[30] Dave SS (2006). Molecular diagnosis of Burkitt's lymphoma. N. Engl. J. Med..

[31] Bethge N (2013). Identification of highly methylated genes across various types of B-cell non-hodgkin lymphoma. PLoS ONE.

[32] Brune V (2008). Origin and pathogenesis of nodular lymphocyte-predominant Hodgkin lymphoma as revealed by global gene expression analysis. J. Exp. Med..

[33] Bakkebo M (2012). SARA is dispensable for functional TGF-beta signaling. FEBS Lett..

[34] Patterson JC (2019). VISAGE reveals a targetable mitotic spindle vulnerability in cancer cells. Cell Syst..

[35] Bai B, Myklebust JH, Walchli S (2020). Gene editing in B-lymphoma cell lines using CRISPR/Cas9 technology. Methods Mol. Biol..

[36] Rosset A, Spadola L, Ratib O (2004). OsiriX: An open-source software for navigating in multidimensional DICOM images. J. Digit. Imaging.

[37] Huse K (2011). Bone morphogenetic proteins inhibit CD40L/IL-21-induced Ig production in human B cells: Differential effects of BMP-6 and BMP-7. Eur. J. Immunol..

[38] Gul N (2019). The MTH1 inhibitor TH588 is a microtubule-modulating agent that eliminates cancer cells by activating the mitotic surveillance pathway. Sci. Rep..

[39] Helleday T (2014). Cancer phenotypic lethality, exemplified by the non-essential MTH1 enzyme being required for cancer survival. Ann. Oncol..

[40] Clarke AR (1993). Thymocyte apoptosis induced by p53-dependent and independent pathways. Nature.

[41] Lowe SW, Ruley HE, Jacks T, Housman DE (1993). p53-dependent apoptosis modulates the cytotoxicity of anticancer agents. Cell.

[42] Stokke T (2000). Oncogenic aberrations in the p53 pathway are associated with a high S phase fraction and poor patient survival in B-cell Non-Hodgkin's lymphoma. Int. J. Cancer.

[43] Fiskvik I (2013). Karyotyping of diffuse large B-cell lymphomas: Loss of 17p is associated with poor patient outcome. Eur. J. Haematol..

[44] Fiskvik I (2015). Combining MYC, BCL2 and TP53 gene and protein expression alterations improves risk stratification in diffuse large B-cell lymphoma. Leuk. Lymphoma.

[45] Ichikawa A (1997). Mutations of the p53 gene as a prognostic factor in aggressive B-cell lymphoma. N. Engl. J. Med..

[46] Sesques P, Johnson NA (2017). Approach to the diagnosis and treatment of high-grade B-cell lymphomas with MYC and BCL2 and/or BCL6 rearrangements. Blood.

[47] Riedell PA, Smith SM (2018). Double hit and double expressors in lymphoma: Definition and treatment. Cancer.

[48] Lin A (2019). Off-target toxicity is a common mechanism of action of cancer drugs undergoing clinical trials. Sci. Transl. Med..

