# Supplementary material for: Karonudib has potent anti-tumor effects in preclinical models of B-cell lymphoma
Source: Sci Rep. 2021 Mar 18;11:6317. doi: 10.1038/s41598-021-85613-8 (PMC7973795; doi:10.1038/s41598-021-85613-8)

Supplemental data

Karonudib has potent anti-tumor effects in preclinical models of B-cell lymphoma

Morten P. Oksvold[1,2], Ulrika Warpman Berglund[3], Helge Gad[3,4], Baoyan Bai[1,2] Trond Stokke[5], Idun Dale Rein[5], Therese Pham[3], Kumar Sanjiv[3], Geir Frode Øy[6], Jens Henrik Norum[7], Erlend B. Smeland[1,2], June H. Myklebust[1,2], Thomas Helleday[3,4] and Thea Kristin Våtsveen[1,2]

[1]Department for Cancer Immunology, Institute for Cancer Research, Oslo University Hospital, Oslo, Norway.

[2]KG Jebsen Centre for B cell malignancies, Faculty of Medicine, University of Oslo, Oslo, Norway.

[3]Department of Oncology and Pathology, Science for Life Laboratory, Karolinska Institutet, Stockholm, Sweden.

[4] Weston Park Cancer Centre, Department of Oncology and Metabolism, University of Sheffield, Sheffield, S10 2RX, United Kingdom

[5]Department of Radiation Biology, Institute for Cancer Research, Oslo University Hospital, Oslo, Norway.

[6] Department of Tumor Biology, Institute for Cancer Research, Oslo University Hospital, Oslo, Norway.

[7]Department of Cancer Genetics, Institute for Cancer Research, Oslo University Hospital, Oslo, Norway.

Running title: Preclinical efficacy of karonudib (TH1579) in B-cell lymphoma

Correspondence: Thea Kristin Våtsveen, Department for Cancer Immunology; Institute for Cancer Research, The Norwegian Radium Hospital, Oslo University Hospital, Ullernschausseen 70, Montebello, 0379 Oslo, Norway. E-mail: thea.kristin.vatsveen@rr-research.no

Supplemental Table 1. Mutational status of *TP53* and *MYC* in the B-cell lymphoma cell lines used in this study sorted from highly to less responsiveness towards karonudib (based on CellTiterGlo data shown in Figure 1A).

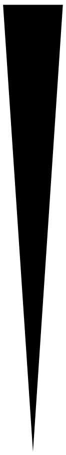

	TP53 mut	*MYC* mut	*MYC* translocation
Will-2	*	*	IGL-MYC
SU-DHL-6	mut	wt	t(8;9)(q24;p13)
BL-41	mut	mut	IGL-MYC
Mino	mut	wt	Overexpression without translocation
U2932	*	mut	
Jeko-1	mut/del	wt	Amplification
Ramos	mut	*	IGH-MYC
DOHH-2	wt	*	IGH-MYC
Granta	del	wt	
Rec-1	mut	*	IGH-MYC
SU-DHL-4	mut	wt	
Raji	mut	mut	IGH-MYC

* mutational status not found in litterature

Supplemental Table 2: GSEA Hallmark gene set analysis

GSEA Hallmark gene sets name	Enrichment Score	Normalized Enrichment Score	Nominal p-val	False Discovery Rate (FDR) q-val	Family wise-error rate(FWER) p-val
MITOTIC SPINDLE	0.53834605	2.1105082	0.0	0.0	0.0
G2M CHECKPOINT	0.47603992	1.8979183	0.0	0.00157203	0.006
SPERMATOGENESIS	0.47970912	1.5647411	0.01417322	0.05185862	0.264
CHOLESTEROL HOMEOSTASIS	0.46559724	1.5107267	0.02907916	0.06879249	0.402
UV RESPONSE DN	0.31867167	1.1182878	0.27769348	0.90039265	1.0
E2F TARGETS	0.27396710	1.0979584	0.24735449	0.82962990	1.0
WNT BETA CATENIN SIGNALING	0.38637772	1.0768290	0.36409396	0.79128290	1.0
TGF BETA SIGNALING	0.32207665	0.9705026	0.49579832	1.0	1.0
NOTCH SIGNALING	0.37129718	0.9450190	0.53159850	1.0	1.0
KRAS SIGNALING DN	0.28912762	0.8620862	0.66611844	1.0	1.0
GLYCOLYSIS	0.22870597	0.8598761	0.75817925	1.0	1.0
ANDROGEN RESPONSE	0.20472237	0.6928398	0.94470050	1.0	1.0
ALLOGRAFT REJECTION	0.18501072	0.6787430	0.98666670	1.0	1.0
MYC TARGETS V2	0.19891097	0.6564656	0.96978850	1.0	1.0
PROTEIN SECRETION	0.17703673	0.6304441	0.98984030	1.0	1.0
MYC TARGETS V1	0.13134864	0.5281278	1.0	1.0	1.0
APICAL SURFACE	0.19738322	0.5055977	0.97590363	0.9976106	1.0

Supplemental Figures:

Supplemental Figure 1

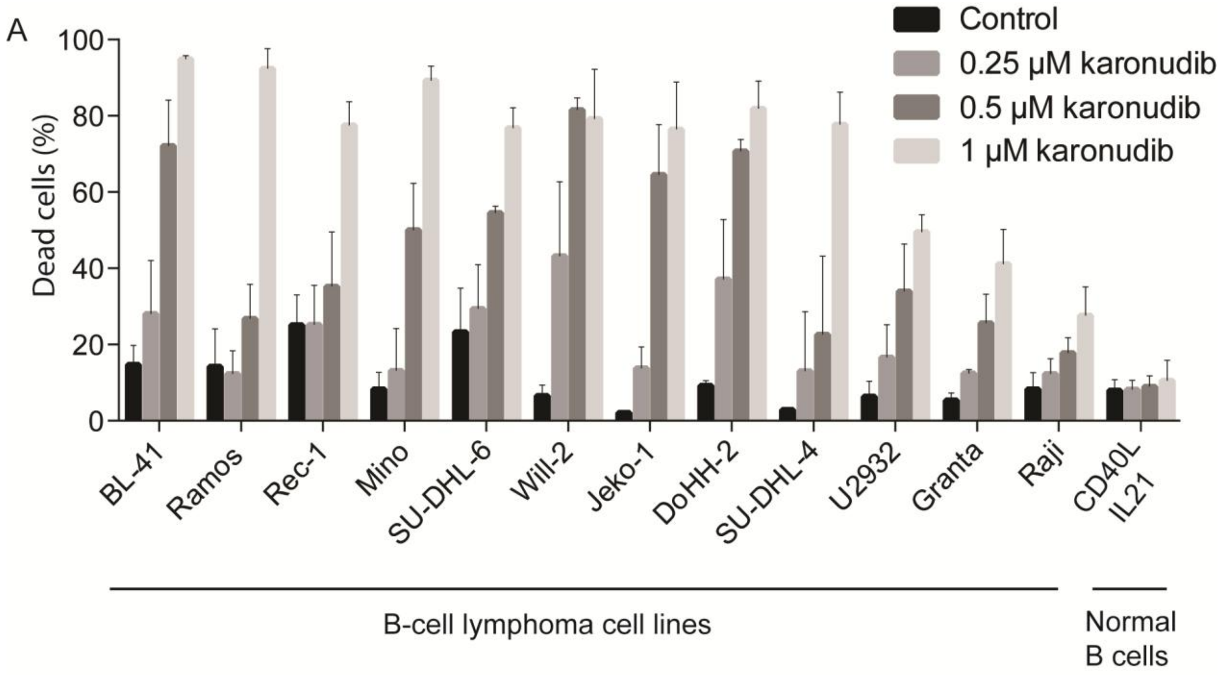

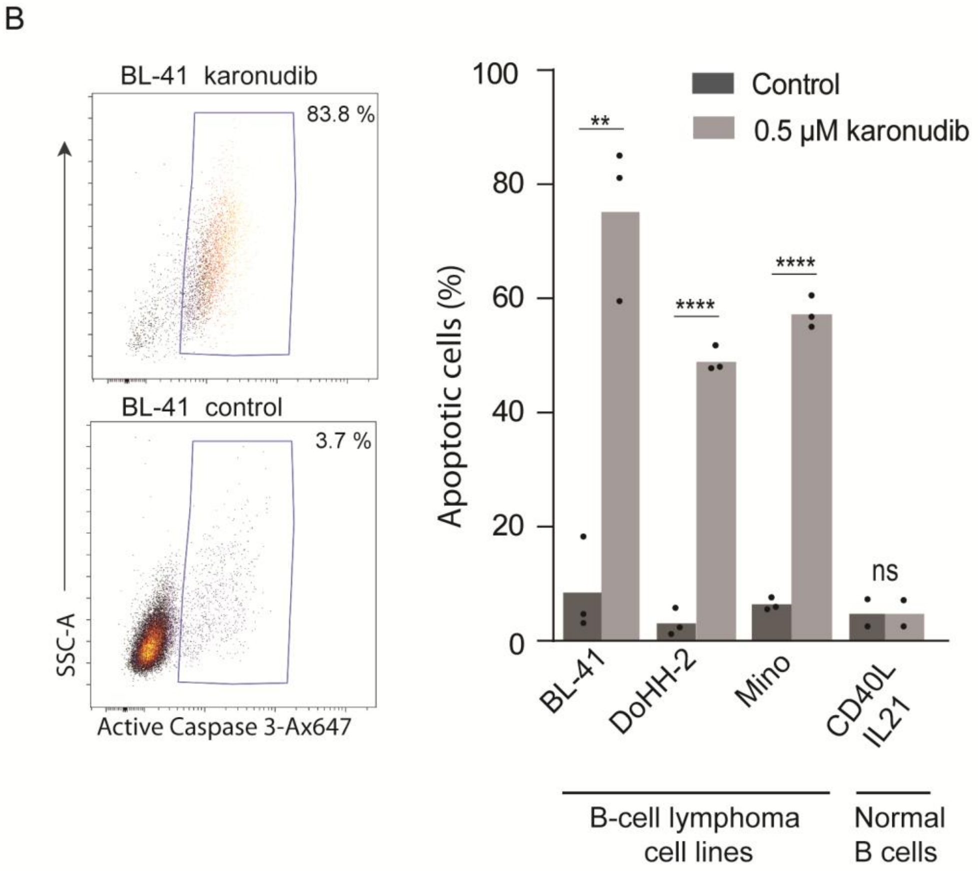

Supplemental Figure 1. Treatment with karonudib induces apoptosis in B-cell lymphoma cell lines, but not in normal B cells. (A) B-cell lymphoma cell lines and normal activated (CD40L/IL21) B cells were exposed to increasing concentrations of karonudib (0.25, 0.5 and 1 µM, 72 h, *n* = 3). Induction of cell death was seen in a dose dependent manner, measured by propidium iodide (PI)-staining. (B) B-cell lymphoma cell lines (*n* = 3) and normal activated B cells (*n* =2) were treated with karonudib (0.5 µM, 24 h) and apoptotic cells were identified by measuring active caspase-3. A and B was assessed by flow cytometry on live and PFA/methanol fixed cells respectively.

Supplemental Figure 2

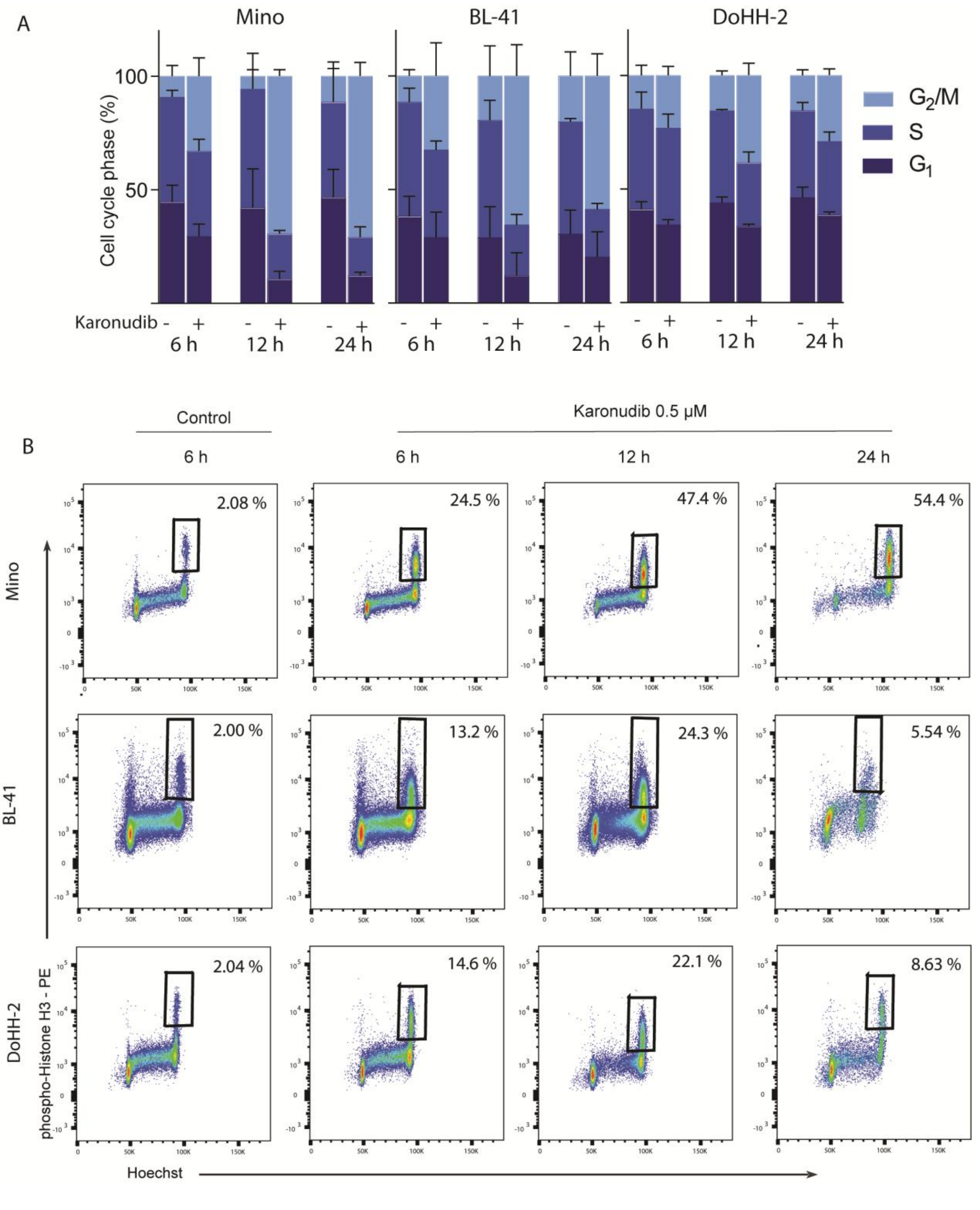

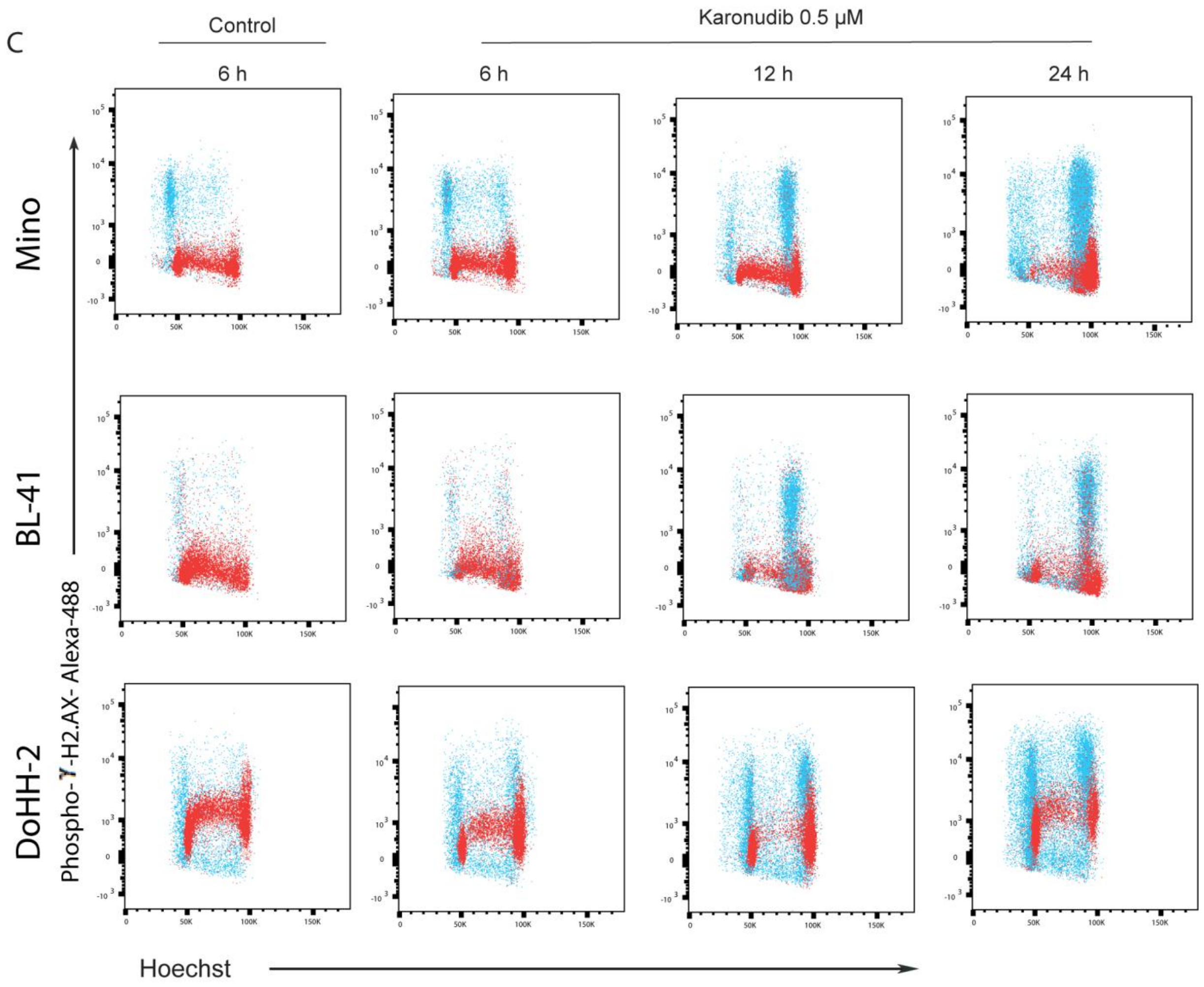

Supplemental Figure 2. (A) Cell cycle profile for the dead cells is shown for untreated control and karonudib treated cells. An increased level of both G_2/M-phase is seen in the karonudib treated cells compared to control (B) Treatment with karonudib induces metaphase arrest, shown by staining for phospho-h istone H3 and with Hoechst. The dot plots show the metaphases in live cell populations. In all three cell lines metaphase arrest was observed after 6 h of drug exposure and was independent of *TP53* mutational status. The metaphase arrest was most pronounced in Mino (>50% of the cells arrested in metaphase after 24 h treatment with karonudib). (C) Phospho-γ-H2AX is shown for live (red cells) and dead (blue cells). There was no increase in the phospho- γ-H2AX in the live cells and the phospho- γ-H2AX staining does not show the "horse shoe shape" as common with DNA-damage, but is mainly expressed in the dead cells. Gating strategy for live and dead cells is presented in the main article (Figure 2B).

Supplemental Figure 3

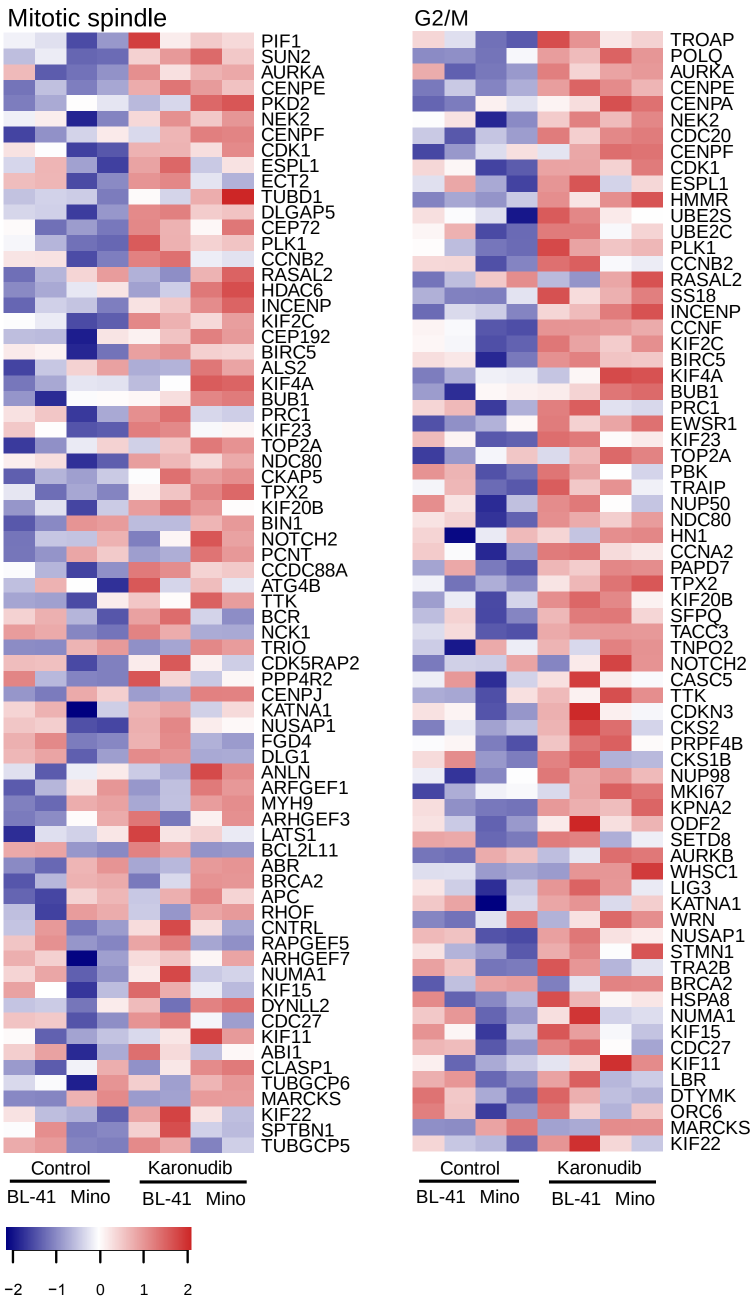

Supplemental Figure 3. Heat map from the gene enrichment profile signatures. BL-41 and Mino was treated with karonudib (0.5 µM, 12 h) and microarray analysis were performed on GeneChip Human Gene 2.0 ST Array (affymetrix). Gene set enrichment analysis was performed using the GSEA software v-3.0 combining both cell line data against predictive gene sets (Hallmark datasets). A thousand permutations were performed to test against control and karonudib treated cells. Mitotic spindle and G2/M was the only two significantly changed datasets (Supplemental table S2). Only genes that contributed to the significant signatures is included in the heat maps.

Figure 4 *NUDT1* CRISPR KO clones

Mino-CAS9 cell line was transfected with guide RNA CATGAAAAAGCGAGGCTTCG or TTCGGGGCCGGCCGGTGGAA (named Sg1 and only shown in supplement). Cells were expanded and cloned out. Primers for Sanger sequencing: Fw:TCTACCCCAGAAAATCACTGGT and Rw:ATCATCAGCTCCACTGAACAGA was used. Sequence were validated using TIDE (Tracking of Indels by DEcomposition, Brinkman *et al*, Nucleic Acids Res. 2014 Dec 16;42(22):e168. doi:10.1093/nar/gku936.) and CRISP-ID (Dehairs, J. *et al.* CRISP-ID: decoding CRISPR mediated indels by Sanger sequencing *Sci. Rep.* 6, 28973; doi: 10.1038/srep28973 (2016).). Mino has three copies of chromosome 7, hence a more complex genotype is formed. Two clones with editing in all 3 alleles of *NUDT1* were chosen for further experiments based on the data from TIDE and CRISP-ID.

A) *NUDT1* KO1

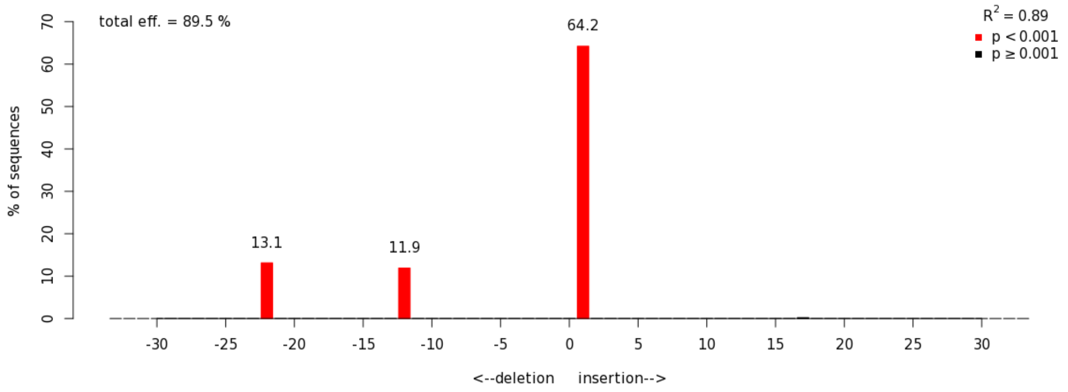

B) *NUDT1* KO3

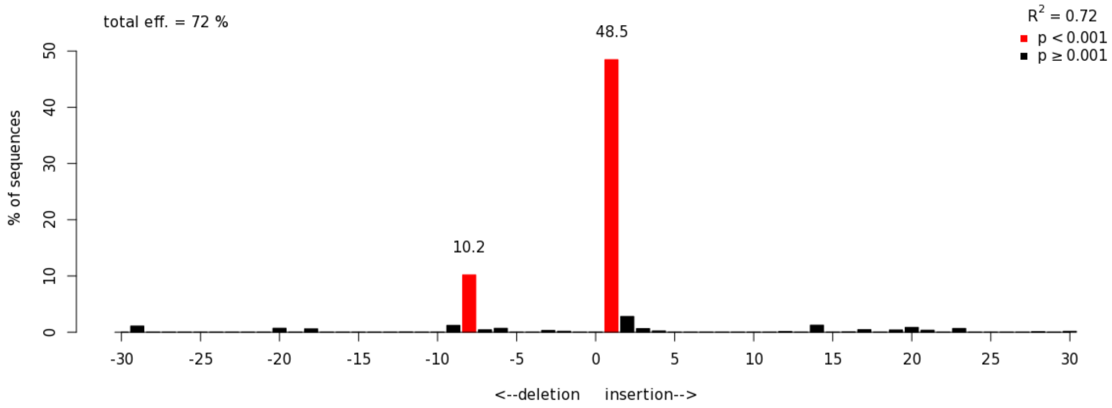

C)

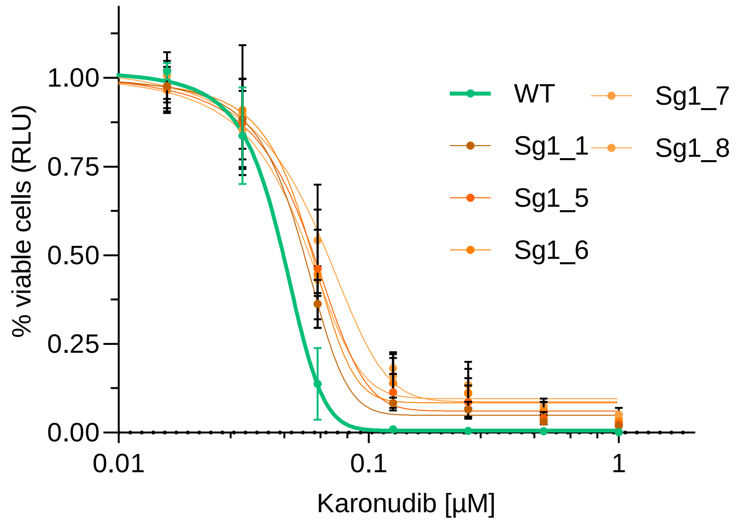

D)

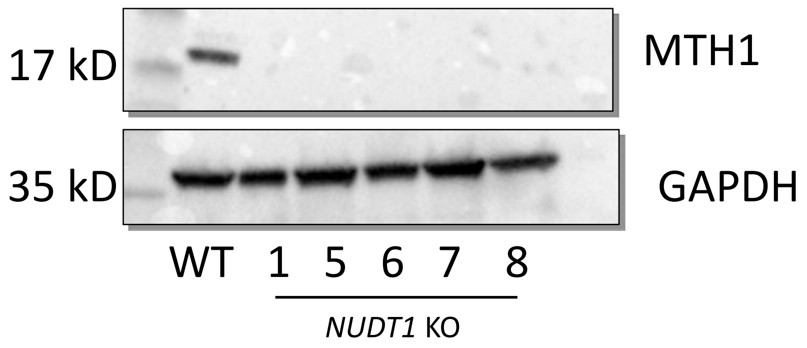

Supplemental Figure 4. Extra information and validation of the *NUDT1* KO clones. A)TIDE plot showing indels in Mino *NUDT1* KO1 and B) *NUDT1* KO3. All indels led to the formation of a stop codon leading to termination of translation of MTH1. C) CellTiterGlo viability data (0.012-1 µM karonudib, 72 h) shown as in Figure 6B. This is clones from another guide-RNA than shown in the main manuscript. A shift for the tolerance to karonudib by the *NUDT1* KO clones is seen similar to the other clones. D) Sg1 clones shown on immunoblots MTH1 Antibody (PA5-96335) polyclonal rabbit from Invitrogen. All clones were tested with both monoclonal antibody from santa cruz (main manuscript and from invitrogen as shown here with the same result).

Supplemental Figure 5

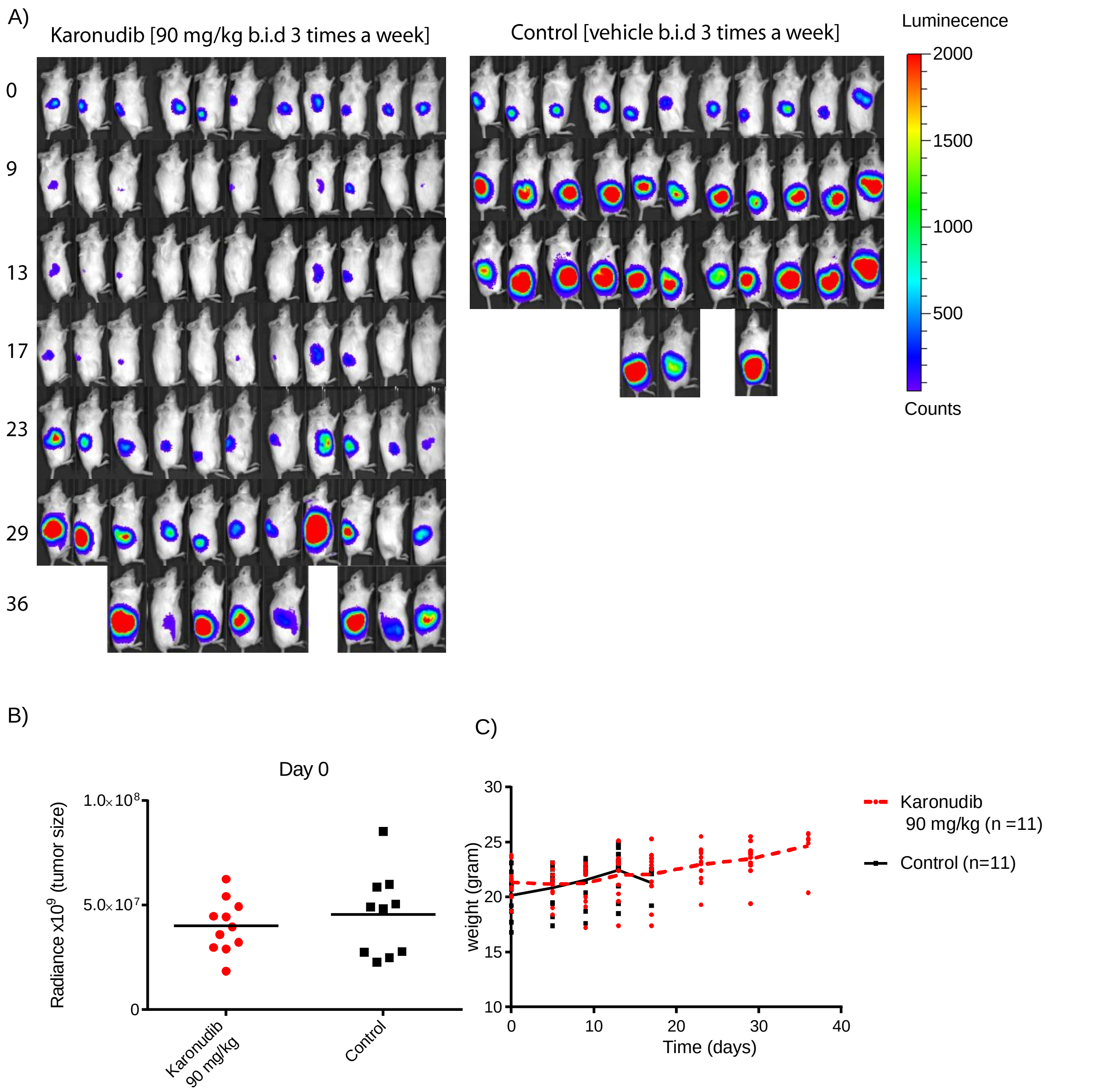

Supplemental Figure 5. Effect of karonudib on tumor growth and weight in BL-41-luc xenograft study. (A) Tumor is visualized by IVIS images for all mice at day 0, 9, 13, 17, 23, 29 and 36. A significant decrease in tumor size in the treated group is seen early (day 9; $P < .0001$), which is consistent at day 13 and 17 ($P < .0001$ for both time points). After treatment was discontinued, tumor reappeared (day 23 compared to day 0, $P = .16$). (B) The distribution of tumor size in mice before treatment start is shown ($P = 0.46$). The mice were evenly distributed based on tumor size in each cage. Mouse number 11 (far right) in the control group was registered as an outlier. In this mouse the tumor material was distributed differently compared to the other mice (more spread). This mouse was considered removed from the study. However, including or removing this outlier does not alter the result of the study and it was therefore included. (C) Karonudib was well tolerated, seen as a steadily overall increase in weight for both control and treatment group. However, mouse number 2 (from left) in the treated group (A), showed weight loss and underwent a

treatment pause on treatment day 4, followed by half drug dosage at day 5 and 6. No increase in tumor volume was observed due to reduced drug dosage in this mouse, and it regained its weight on the lower dose of karonudib.

Supplemental Figure 6

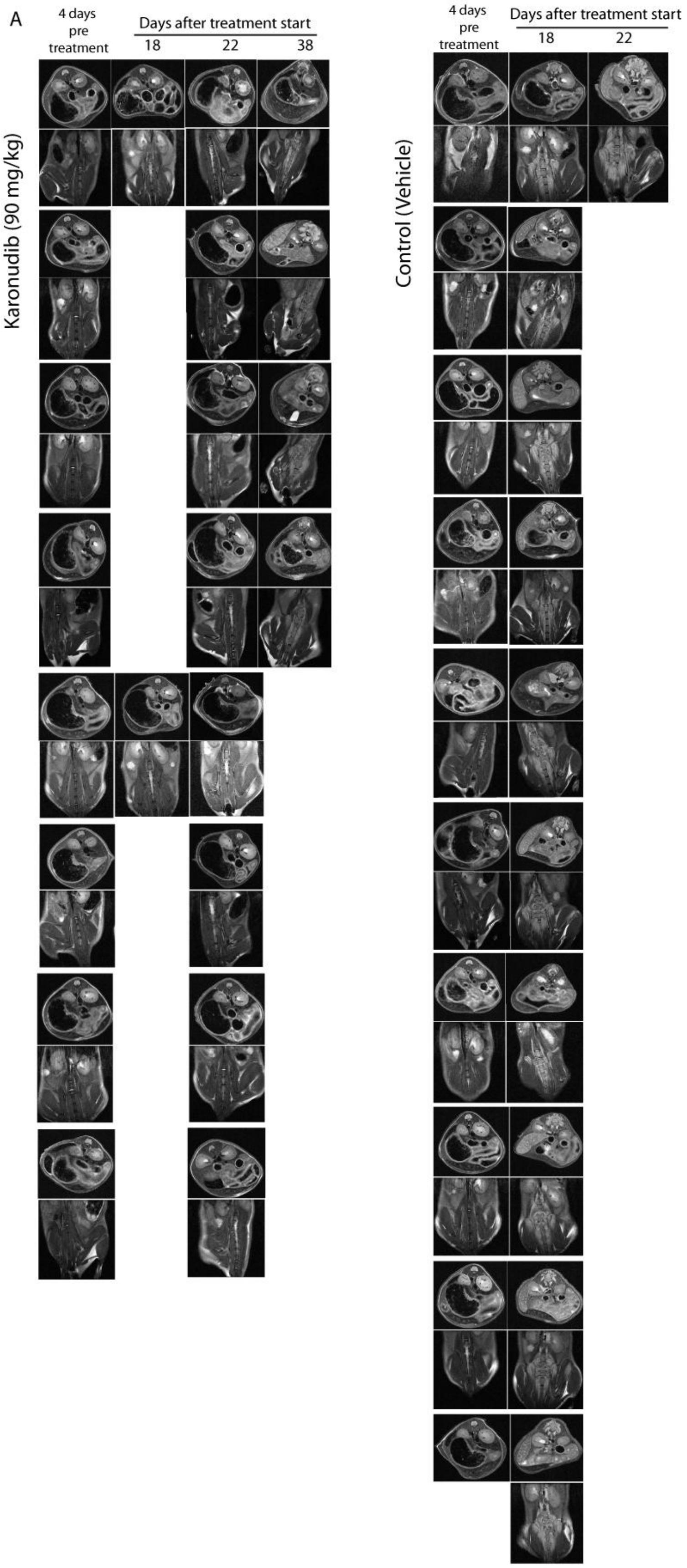

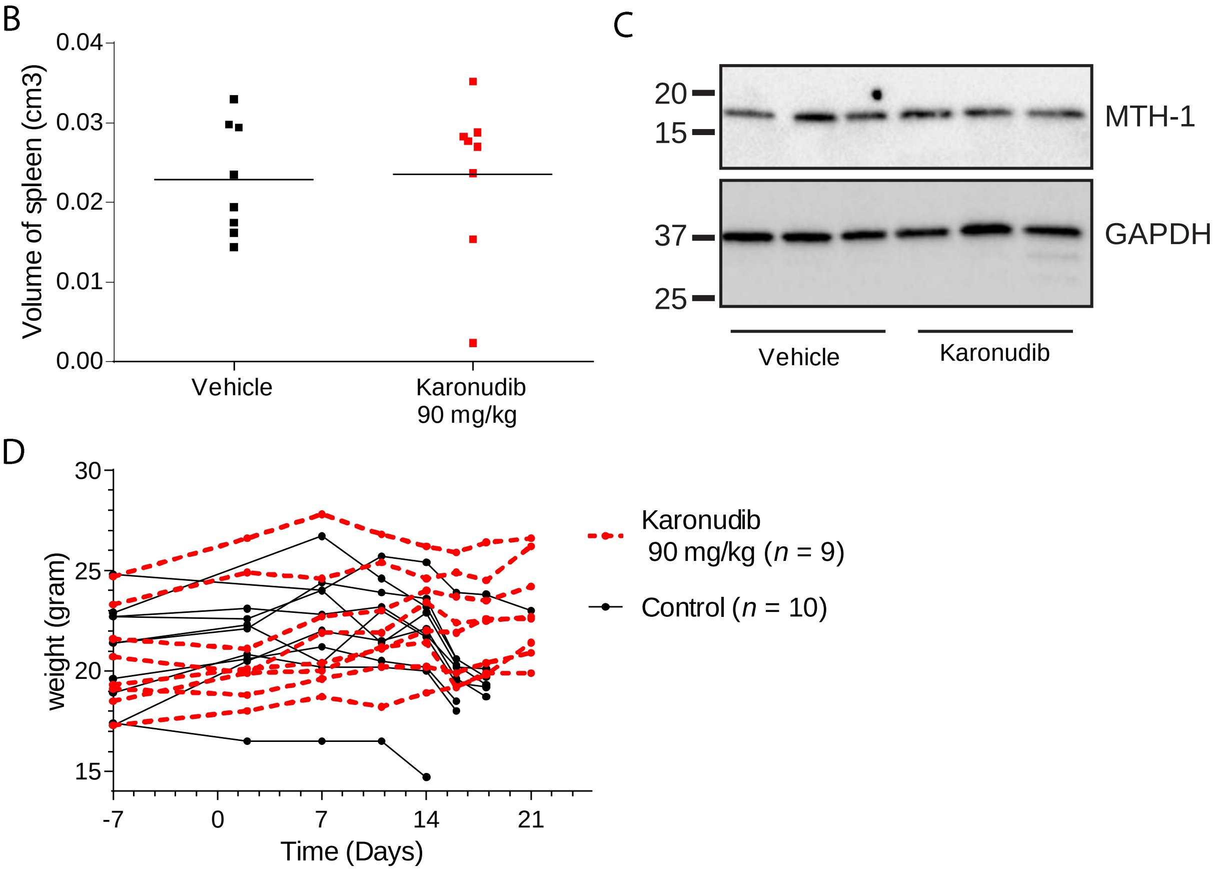

Supplemental Figure 6. Karonudib shows potent inhibition of growth in a preclinical PDX-model DFBL-49659-V2 from a DLBCL-ABC patient. A) All MRI scans from the xenograft study. Tumors are located in spleen and bone marrow. In the last scan taken when mice showed clinical symptoms, edema around the bone marrow was seen. For the karonudib treated mice that relapsed after treatment stop the edema was also seen, although not all mice had an enlarged spleen. B) The tumor volume of the spleens measured four days before treatment start. C) MTH1 level in tumor from three vehicle treated mice and three karonudib treated mice after relapse. The karonudib treated tumors were harvested two weeks after treatment stop, and the levels of MTH1 is unchanged. D) The weight of the mice during the course of treatment. The vehicle treated mice lost weight when the tumor growth escalated. Mice treated with karonudib had a steady increase in weight throughout the treatment period.

Supplemental Figure 7

Whole Western Immunoblots. Manuscript figure is indicated above the immunoblots

Figure 1

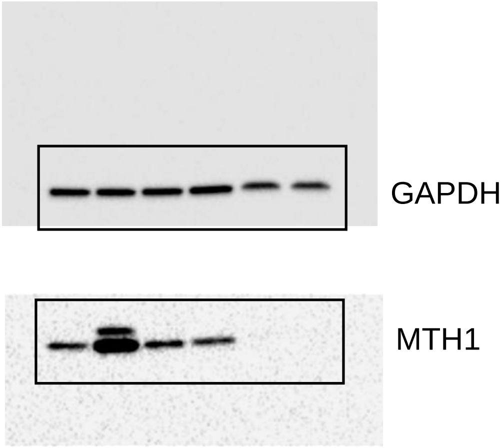

Figure 3

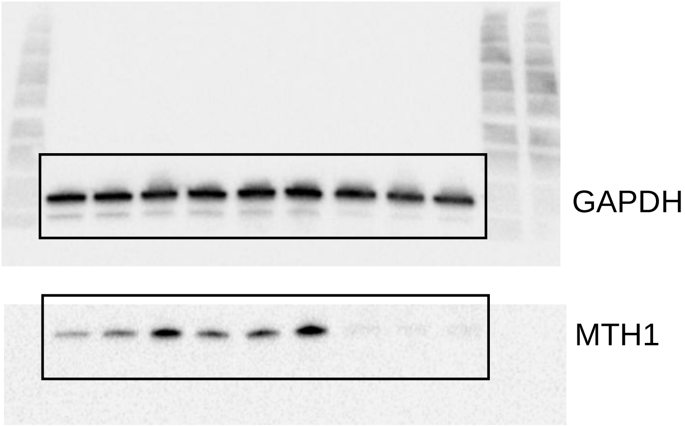

Figure 4

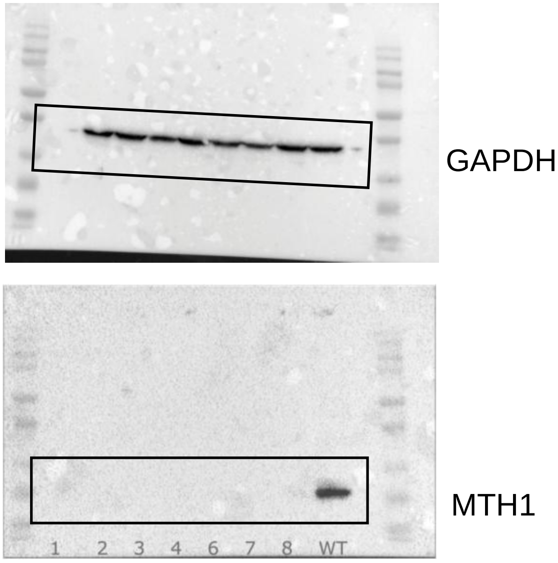

Supplemental figure S4

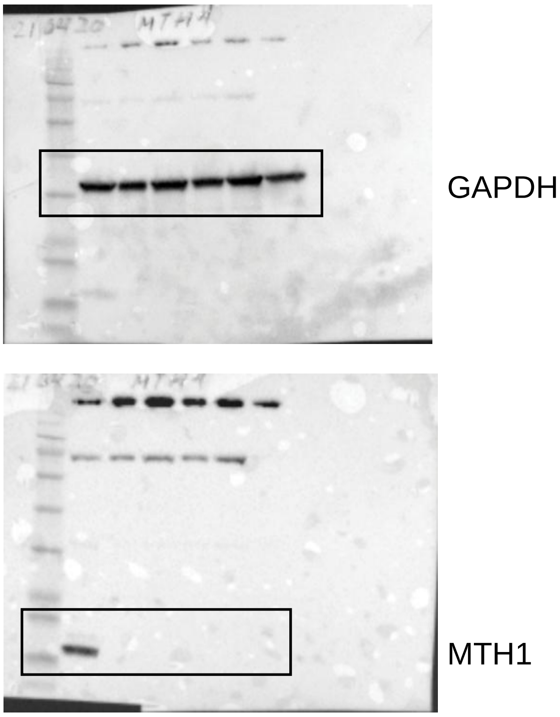

Supplemental Figure S6

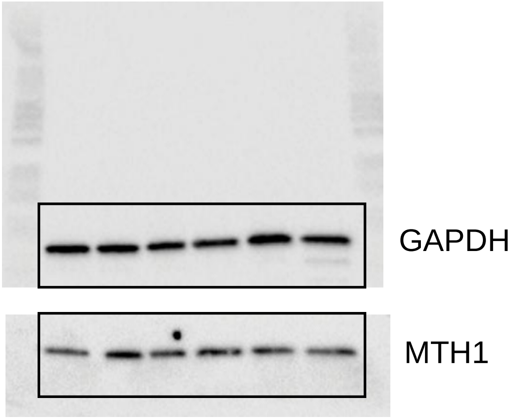

Supplement: Supplementary file 1 — Supplementary Information. [file 41598_2021_85613_MOESM1_ESM.pdf]
